# Disability and Therapeutic Response in Paediatric Neuromyelitis Optica Spectrum Disorder: A Case Series from Iran

**Published:** 2019

**Authors:** Seyed Mohammad BAGHBANIAN, Mohammad Ali SAHRAIAN, Abdorreza NASER MOGHADASI, Nasrin ASGARI

**Affiliations:** 1 Neurology Department; Booalisina Hospital, Mazandaran University of Medical Sciences; Sari, Iran; 2 Neurology Department, MS Research Centre, Neuroscience institute, Tehran University of Medical Sciences, Tehran, Iran; 3 Institutes of Regional Health Research and Molecular Medicine, University of Southern Denmark, J.B. Winsloewsvej 25.2, 5000 Odense C, Denmark

**Keywords:** Neuromyelitis optica spectrum disorder, Paediatric, Disability, Treatment

## Abstract

**Objectives:**

The characteristics of paediatric neuromyelitis optica spectrum disorder (NMOSD) may indicate the degree of disability and identify factors that predict the response to treatment.

**Materials & Methods:**

Among 114 NMOSD patients in an acquired demyelinating syndromes registry at the Sina Hospital, in Tehran, Iran, 10 paediatric NMOSD patients with longitudinal follow-up from 2005 to 2016 were retrospectively identified. The median time between disease onset and diagnosis was 18 months (range 1-108 months).

**Results:**

All patients had a relapsing course, which resulted in disability in six with severe visual impairment and functional blindness in one and impaired ambulation in five patients during follow-up. Azathioprine (AZA) was first drug of choice for prophylaxis, but in five patients new attacks occurred and therapy was switched to rituximab (RTX) with no further relapses after median two years (range 1-3 y) follow-up.

**Conclusion:**

Paediatric onset of NMOSD was associated with severe attacks and poor response in 50 % of cases to AZA, RTX seemed to decrease the relapse rate.

## Introduction

Neuromyelitis optica spectrum disorder (NMOSD) is a rare autoimmune inflammatory disease of the central nervous system (1). In most cases, an autoantibody to the astrocytic water channel protein aquaporin-4 (AQP4-IgG) can be detected (2). AQP4-IgG causes complement-dependent astrocyte injury, inflammation, and consequent demyelination besides axonal damage (2, 3). A high relapse rate and disability accumulation in NMOSD can cause severe neurological disabilities, which can potentially be prevented or modified by appropriate treatment (3). 

An early onset of NMOSD is defined as disease onset prior to 18 years (y)of age (4). The paediatric onset of NMOSD comprises 4% of all cases (4). Paediatric NMOSD rarely occurs before the age of nine, with a variable median age (5, 6). The Paediatric Working Group for Neuromyelitis optica spectrum disorder (NMOSD) has stated that adult consensus criteria are applicable to paediatric patients (1). However, some differences have been noted such as a lower female–male ratio (3:1) compared with adults (9:1), more cases with a monophasic course, and less specificity of a longitudinally extensive transverse myelitis (LETM) lesion on spinal cord MRI (7). 

We aimed to review and analyse detailed data of paediatric onset of NMOSD cases including clinical manifestations and treatment responses, the data underscore the presence of severe disability in paediatric patients and may assist in the identification of factors that predict the response to treatment.

## Patients and Methods

***Patients***

Of the 114 NMOSD patients registered in the Sina Hospital NMOSD Clinic of the Tehran University of Medical Sciences, Tehran, Iran, 10 showed paediatric onset (< 18 y of age). These cases were diagnosed from 2005 to 2016. Their clinical symptoms, laboratory data and MRI findings were reviewed and questionnaires were used to provide long-term retrospective data. All patients had been diagnosed according to last international consensus criteria of NMOSD (1).

***Laboratory methods***

Determination of AQP4-IgG was carried out using an indirect immunofluorescence (IFA) assay with HEK293 cells transfected with recombinant human full-length *AQP4* gene starting at a 1:10 dilution (Euroimmun, Lübeck, Germany) (8).

***Ethics approval***

Oral and written informed consent was obtained from all participants. The study was carried out in accordance with the Tehran University of Medical Sciences Ethical Committee guidelines.

***Statistical analysis***

Statistical differences were tested using the Wilcoxon rank sum test for two-group comparisons and the Fisher exact test for group comparisons of categorical variables. A P-value < 0.05 was considered significant.

**Table 1:** Demographic and clinical characteristics of the patients

Variable	Male	Female
Total number	2	8
Median age at onset/year-old	11(range 9-13)	13.5 (range 8-17)
Median duration of diseases before diagnosis/months	9 (range 6-12 months)	24(range 1-108 months)
Positive anti-AQP4 Ab	1	5
Abnormal brain MRI	1	6
Cervical LETM/patients	0	1
Thoracic LETM/patients	1	2
Cervicothoracic LETM/patients	1	3
Median length of LETM/Segment	9 (range 7-11 segment)	7.5(range 3-12 segment)

LETM = Longitudinal extensive transverse myelitis

**Table 2:** Annualized relapse rate before and after preventive therapy

ARR/Patient/year	P1	P2	P3	P4	P5	P6	P7	P8	P9	P10
ARR before preventive therapy	0.6	0.8	1	1	2	2	1	1	1.5	1
ARR after azathioprine	2	2	0	1	0	0	0	1	0	1
ARR after rituximab	0	0		0				0		0

ARR = Annual relapse rate

## Results

Demographic data and clinical course

The demographic and clinical characteristics of the patients are depicted in Table 1. Ten patients (seven AQP4-IgG positive) aged under 18 yr at disease onset were included. The female–male ratio was 4:1. The median age at disease onset was 13 y (range: 8–17 y). The median age of the patients at the disease diagnosis was 14 yr (range: 10-26 yr). Thirty-four relapses were recorded for all patients. 

Clinical presentation

The initial presentation in four of the patients was optic neuritis (ON); in one, simultaneous ON/ transverse myelitis (TM); in three, LETM; and in two, area postrema syndrome (APS). ON in these cases was characterised by severe visual acuity, in three with unilateral ON, and in one with sequential bilateral ON. Visual acuity was lowered to less or equal to 20/200 in two patients and in one patient ended up with a permanent unilateral blindness despite intensive treatment. The disease followed a relapsing course in all 10 patients. The median time between disease onset and diagnosis was 18 months (range 1-108 months).

The second attack was ON (seven) and TM (three), all with LETM. During follow-up, acute TM occurred in two patients. Acute TM/LETM caused tetraparesis in two patients, paraparesis in seven patients, dysesthesia in all patients. Patients were followed for median two y (range 2-4 y) after diagnosis.

Neuroimaging findings

Brain MRIs after the first attack were normal in seven cases. The three cases with abnormal brain MRI at the first presentation showed nonspecific scattered white matter subcortical lesions (one patient), area postrema involvement (one patient), and diencephalic lesion (one patient). The brain MRI follow-up one year later was normal in seven and nonspecific in three patients. 

Spinal cord MRI in two patients demonstrated cervical LETM reaching into the brainstem with area postrema involvement, while one patient showed thoracic LETM at the onset of NMOSD. During follow-up, eight patients had LETM. Notably, the median length of the LETM was 9 (range 7-11) vertebral body heights in males and 7.5 (range 3-12) in females. LETM was noted in the cervical MRIs of one patients, the thoracic MRIs of three patients, and the cervicothoracic MRIs of four patients. 

Lab data

AQP4-IgG was positive in seven patients with ELISA and IIF assays. Anticardiolipin antibodies (IgG and IgM), antinuclear antibodies (ANA), anti-dsDNA antibodies, anti-Sjögren's-syndrome-related antigens A and B, also called anti-Ro and anti-La angiotensin converting enzyme (ACE), and serum human T-lymphotropic virus (HTLV) 1 and 2 antibodies were negative in all patients and no coexisting autoimmunity or family history of autoimmune disease was observed. 

Treatment

Initial acute attack was treated with high-dose intravenous methylprednisolone (IVMP) (30 mg/kg/d for 5 days up to a maximum of 1000 mg daily) (9). In 50% (five) of the patients, there was no improvement from pulse therapy alone and plasma exchange (PLEX) was introduced. Despite PLEX, visual acuity decreased in four patients to hand motion. Full recovery was achieved by PLEX in one case after the IVMP failure.

All patients received azathioprine (AZA) (2–3 mg/kg) (9) as the first line of therapy for relapse prevention. Although AZA was well tolerated, it did not prevent further exacerbations in five patients every six months over the next two years. The median annual relapse rate changed from 1 (range 0.6-2) before preventive therapy to 0.5 (range 0-2) after the therapy with azathioprine (Table 2). Five of the patients had their treatment switched to rituximab (RTX) because of new relapse on treatment with azathioprine, while no further relapse occurred with RTX in these five patients within two years (Table 2). RTX was administered as 1 g given IV 2 weeks apart and then repeated every 6 months. No severe complications occurred with RTX. The median expanded disability status scale (EDSS) before preventive therapy was 3 (0-5 range) and decreased to 2.5 (range 0-5) after preventive therapy (Table 3). 

## Discussion

This retrospective case series with longitudinal follow-up aimed to describe paediatric NMOSD including clinical, neuroimaging characteristics, and treatment responses. The data indicated that the onset of NMOSD during childhood was associated with severe attacks with irreversible disability despite intensive treatment including PLEX. Immunosuppressive treatment seemingly decreased the relapse rate, however, a poor response to AZA was observed in half of the cases, leading to the use of RTX, being a more potent therapy. Overall, these findings emphasize the importance of patient identification, which may lead to early and effective treatment and define key areas for future research. 

A heterogeneous pattern of clinical paediatric NMOSD manifestations was observed, including ON, TM/LETM, and brainstems lesions. LETM tended to occur during the course, not at onset, of paediatric NMOSD. The disease followed a relapsing course in all patients and resulted in disability with severe visual impairment or functional blindness; it also impaired ambulation during follow-up. In all the 10 patients, immunosuppressive therapy was started with AZA. Five of the patients tolerated AZA well with no further attacks, but in five other patients new attacks occurred and immunosuppressive therapy was switched to RTX. No further relapses occurred for two years in the five patients on treatment with RTX. 

RTX is an anti-CD20 chimeric monoclonal antibody that depletes B cells and is used in autoimmune and inflammatory CNS disorders including NMOSD (10). In adult NMOSD one prospective and 3 retrospective, RTX reduced the annualized relapse rate (10). One prospective paediatric study of RTX reported that RTX was effective in relapse prevention (11). In the present study after starting preventive treatment, the median annual relapse rate decreased. Although the lowering of EDSS was not significant after preventive treatment probably because of the small sample size, the median relapse rate decreased significantly. The current case series is the first Iranian report on RTX treatment of paediatric NMOSD. 

**In Conclusion,** paediatric onset of NMOSD does occur. As a consequence, NMOSD may be considered a differential diagnosis for paediatric MS. The clinical phenotype was compatible with other reports of paediatric NMOSD cases. Disability occurred incrementally as a result of attacks. It seems that the immunosuppressive treatment for paediatric NMOSD patients may decrease the relapse rate and that RTX is a superior treatment option. Larger studies are required to further evaluate long-term disability in this regard.
